# Insight into the Properties of Plasmonic Au/TiO_2_ Activated by O_2_/Ar Plasma

**DOI:** 10.3390/nano12010106

**Published:** 2021-12-29

**Authors:** Xiaoqing Deng, Yu Ding, Xiaobing Wang, Xiaojing Jia, Shuo Zhang, Xiang Li

**Affiliations:** 1 School of Chemistry and Environmental Engineering, Yangtze University, Jingzhou 434023, China; dxq_j18@163.com (X.D.); ding50_live@163.com (Y.D.); Xicewang@gmail.com (X.W.); jsn070924@163.com (X.J.); 2 School of Environmental Science and Engineering, Hebei University of Science and Technology, Shijiazhuang 050018, China; zhangsure23@163.com; 3 College of Mechanical Engineering, Yangtze University, Jingzhou 434023, China

**Keywords:** CO oxidation, O_2_/Ar plasma, Au/TiO_2_, plasma activation

## Abstract

The performance of CO oxidation over plasmonic Au/TiO_2_ photocatalysts is largely determined by the electric discharge characteristics and physicochemical properties of discharge gas. To explore the activation mechanism of Au/TiO_2_, an O_2_ and Ar mixture gas as a discharge gas was employed to activate Au/TiO_2_. The photocatalytic activity in CO oxidation over activated Au/TiO_2_ was obtained, and the electric discharge characteristics, Au nanoparticle size, surface chemical state, optical property and CO chemisorption were thoroughly characterized. As the O_2_ content increases from 10% to 50%, the amplitude of the current pulses increases, but the number of pulses and the discharge power decrease. The photocatalytic activity of Au/TiO_2_ rises rapidly at first and then remains constant at 75% when the O_2_ content is above 50%. Compared with the discharge gas of 10% and 30% O_2_/Ar, the sample activated by 50% O_2_/Ar plasma possesses less metallic Au and more surface oxygen species and carbonate species by X-ray photoelectron spectroscopy, which is consistent with UV-vis diffuse reflectance spectra and CO chemisorption. The CO chemisorption capacities of the activated samples are the same at a long exposure time due to the approximate Au nanoparticle size observed by transmission electron microscopy. An increase in carbonate species generated from the oxygen species on the surface of TiO_2_ is discovered.

## 1. Introduction

Au/TiO_2_, one of the most promising visible-light photocatalysts, can strongly interact with resonant photons in a large faction of the abundant solar flux due to its strong local surface plasmon resonance (LSPR) [1,2,3]. In one of the widely accepted mechanisms, incoming photons and plasmon resonance increase the energy of electrons of Au nanoparticles. These electrons can thus be injected into the conductance band of TiO_2_ and take part in reactions, leaving a hole behind [4]. This mechanism is analogous to dye sensitization, but noble metals such as Au nanoparticles show more stability and 10^5^ larger charge carrier mobilities than typical dye molecules [5]. Due to its advantages, Au/TiO_2_ has been widely used as a plasmonic catalyst for water splitting [6,7,8], dye degradation [9,10] and indoor air purification [11,12,13,14] in recent decades. The performance of Au nanoparticles significantly depends on the particle size, which is determined by the conditions of activation. When the size of Au nanoparticles decreases from 3.3 to 2.2 nm, the coordinatively unsaturated sites, such as corners and edges, increase from 23% to 33% [15]. Air calcination, as a traditional method, is usually adopted for activation. This method has a serious drawback in that Au nanoparticles suffer from the risk of sintering and structural collapse caused by high temperatures [16]. More secure and efficient activation methods are urgently necessary.

Recently, cold plasma has been widely considered as a promising activation method [17,18]. The gas temperature of cold plasmas, such as DBD, JET, SDBD, etc., is determined by that of heavy ions and other reactive species, which depends on the gas, such as oxygen, nitrogen atom, NO, etc. The gas temperature is much lower than the electron temperature (1~10 eV) and even close to room temperature [19]. In this case, Au particles on the activated Au/TiO_2_ catalyst would be highly dispersed rather than aggregated. In addition, cold plasma is rich in high-energy electrons, metastable particles and active radicals. These particles can fully contact the surface of the catalyst to achieve surface modification [20,21,22,23,24]. A suitable discharge atmosphere is one of the most important factors in the plasma activation of Au/TiO_2_ [25,26,27,28]. The common discharge gases are H_2_, Ar, O_2_ and N_2_. Ar plasma can reduce noble metal nanoparticles and generally presents a greater uniformity and energy efficiency [29,30]. However, excellent discharge properties bring improvement in the efficiency but have an insignificant influence on the activity of catalysts. Ar atmosphere is inferior to other oxidizing atmospheres, such as O_2_, for the activation of Au nanoparticles. O_2_ plasma has the ability to enhance the catalytic performance by unique features such as a lower Au nanoparticle size, plenty of surface oxygen, numerous low-coordinated metallic Au and restructuring of the cationic species [27,31,32,33]. O_2_/Ar plasma is predicted to combine the advantages of Ar plasma and O_2_ plasma. The investigation of photocatalytic activity on Au/TiO_2_ activated by plasmas at various O_2_/Ar ratios would provide insight into the catalytic properties. The relationship between the O_2_/Ar ratio and physicochemical properties of activated Au/TiO_2_ also needs to be explored.

In this work, plasmas at various O_2_/Ar ratios were adopted to activate Au/TiO_2_. The photocatalytic activity over Au/TiO_2_ was valued via CO oxidation under visible-light irradiation. The discharge parameters, Au nanoparticle size, optical properties, surface chemical state and CO chemisorption were analyzed. Moreover, the role of O_2_ in O_2_/Ar plasma during Au/TiO_2_ activation is also discussed.

## 2. Experimental Section

### 2.1. Preparation of Au/TiO_2_ Photocatalysts

The preparation of Au/TiO_2_ photocatalysts was performed by a modified impregnation method, as described in a previous paper [31]. In brief, 1.0 g of P25 (Degussa, Frankfurt, Germany) powder used as the TiO_2_ support was impregnated with 2.2 mL of 2.43 × 10^−2^ mol/L HAuCl_4_ aqueous solution. Then, it was aged for 12 h at room temperature in the dark. Ammonia solution and deionized water were used to wash the powder twice to remove Cl^−^. After washing and filtering, the cake was dried at 80 °C for 8 h. The as-prepared Au/TiO_2_ powder was obtained. The actual Au content was approximately 0.90 wt.%, as determined by inductively coupled plasma atomic emission spectroscopy (ICP-AES, Optima 2000DV, New Brunswick, NJ, USA). The coating of Au/TiO_2_ was obtained for photocatalytic evaluation. The as-prepared Au/TiO_2_ powder (10 mg) was added to 1 mL of deionized water and sonicated for 15 min. The slurry was coated by the dip-coating method on a 25 mm (L) × 25 mm (W) × 1 mm (T) glass substrate and dried at 80 °C for 0.5 h. The weight of the Au/TiO_2_ coating was 15 ± 2 mg.

### 2.2. Plasma Activation and Measurement of Electric Discharge Characteristics

**Figure 1:**
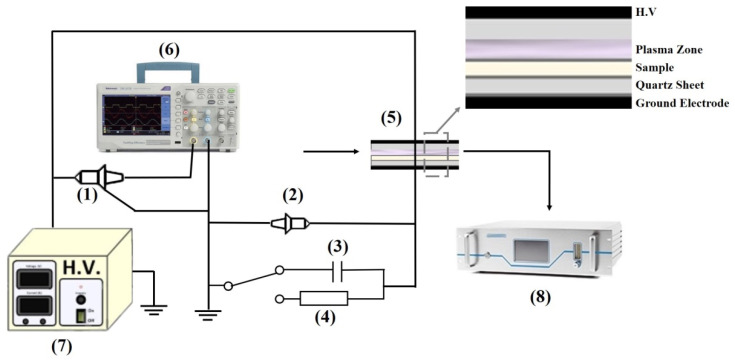
Circuit schematic diagram of electrical parameter measurements in plasma. (1) H.V. probe, (2) passive probe, (3) capacitance, (4) resistance, (5) reactor, (6) oscilloscope, (7) AC high-voltage, (8) -CO_x_ analyzer.

In this paper, plasma was generated by a homemade dielectric barrier discharge at atmospheric pressure to activate Au/TiO_2_ photocatalysts. The setup scheme is shown in Figure 1. The high-voltage electrode and the ground electrode were covered with aluminum foil and stuck to the upper and lower quartz sheets. The gap between the two quartz sheets was 2 mm. The plasma generator was a sinusoidal AC high-voltage generator with a frequency of 1.8 kHz. The input power was a constant of 5 W under various discharge gases and measured by a wattmeter (D51, 0–75 W; HY, Harbin, China). The discharge gas was a mixture of Ar and O_2_, each of which was monitored by flow mass control. The total flow rate during the plasma was kept at 100 mL/min. Plasma activation over all samples was conducted for 30 min.

A circuit schematic diagram of electric parameter measurements in DBD plasma is shown in Figure 1. The power meter was accessed on the primary side of the power transformer, and the input power was measured. The discharge voltage was obtained by a high-voltage probe (1000:1), and the discharge current was calculated by measuring the voltage on the sampling resistor, for which the resistor value was 50 Ω. The discharge power was measured by the Lissajous pattern method.

### 2.3. Photocatalytic Evaluation

The photocatalytic evaluation was carried out under visible light in a continuous flow photocatalytic oxidation reactor. A 300 W X-lamp was employed as the visible-light source, and its wavelength was cut by a 420 nm cutoff filter. The coating sample was placed in the photocatalytic reactor and irradiated through a quartz window (75 mm (L) × 25 mm (W)). The reactant gas was synthetic air (80% N_2_ + 20% O_2_) containing approximately 500 ppm CO with a flow rate of 150 mL/min. A CO_x_ analyzer (TY-6310, Tianyu Intelligent Control, Wuhan, China) was used to measure CO and CO_2_ concentrations. Photocatalytic oxidation can be found in detail in a previous study [31]. The reaction time was approximately 40 min when the CO_2_ concentration reached to a constant. The CO conversion was defined as follows:

(1) $$X_{CO}\left(\%\right)=\frac{C_{CO}^{in}-C_{CO}^{out}}{C_{CO}^{in}}\times 100\%$$

where $C_{CO}^{in}$ and $C_{CO}^{out}$ represent the concentrations of the CO inlet and outlet gases, respectively.

### 2.4. Photocatalyst Characterization

The particle size of samples was observed by transmission electron microscopy (TEM, Tecnai G220 S-Twin, FEI, Hillsboro, OR, USA), where 20 mg of Au/TiO_2_ powder was added into 5 mL of ethanol and dispersed by ultrasound for 15 min. Then, 5 drops of the suspension were dripped on a copper grid with carbon polymer and dried at room temperature for measurement. Chemical binding states and compositions of samples were investigated by X-ray photoelectron spectroscopy (XPS, ESCALAN250, Thermo VG, Boston, MA, USA) using a monochromatized Al K*α* (1486.6 eV) X-ray source. All binding energies are referenced to the C 1s peak at 284.6 eV. Diffuse reflectance UV-visible spectra (UV-vis DRS) of samples in a 200–800 nm range were recorded using a spectrophotometer (V–550, JASCO, Tokyo, Japan). Barium sulfate was used as a background reference. The in situ diffuse reflectance infrared Fourier transform (DRIFT) spectra of CO adsorption were recorded by an FT-IR spectrometer (is50, Thermoscientific, Boston, MA, USA) with a MCT detector at a resolution of 4 cm^−1^. The range of the wavenumber was from 4000 to 1000 cm^−1^. N_2_ as a purging gas at a flow rate of 100 mL/min was used to pretreat Au/TiO_2_ in a DRIFT cell at 80 °C. When the DRIFT cell cooled down to room temperature, the background spectrum was recorded. About 1000 ppm CO/N_2_ at the same flow rate was switched to the DRIFT cell. Then, spectra of CO adsorption were recorded.

## 3. Results and Discussion

### 3.1. Electrical Discharge Characteristics

**Figure 2:**
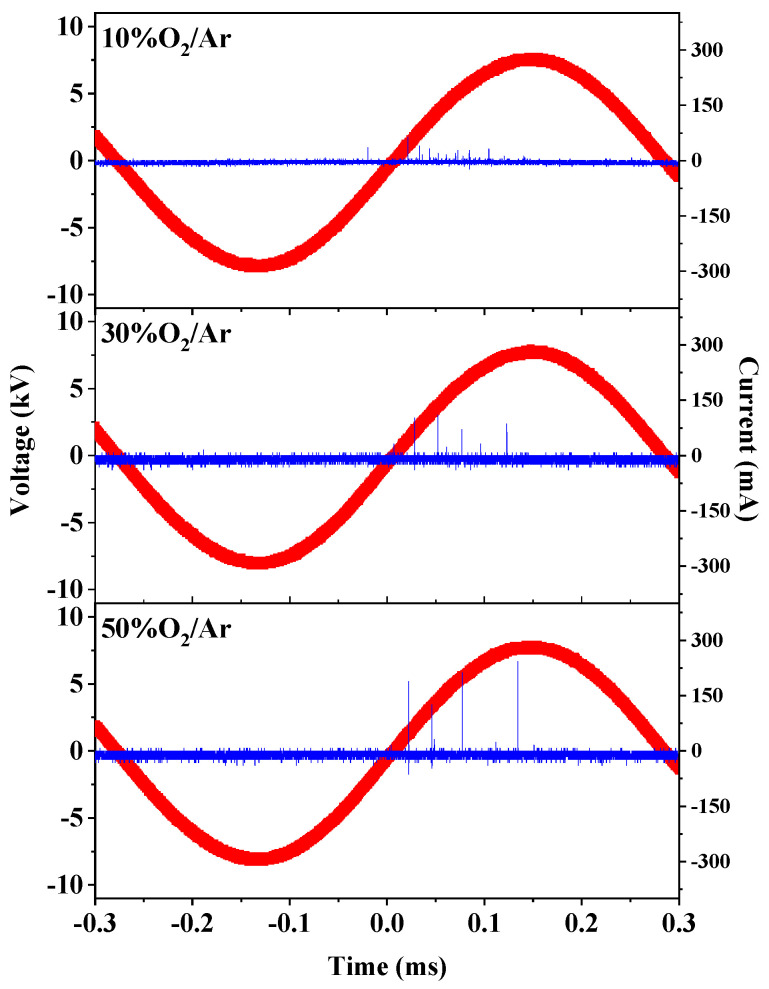
Waveforms of discharge voltage and discharge current at various O_2_ contents in O_2_/Ar discharge gas.

**Figure 3:**
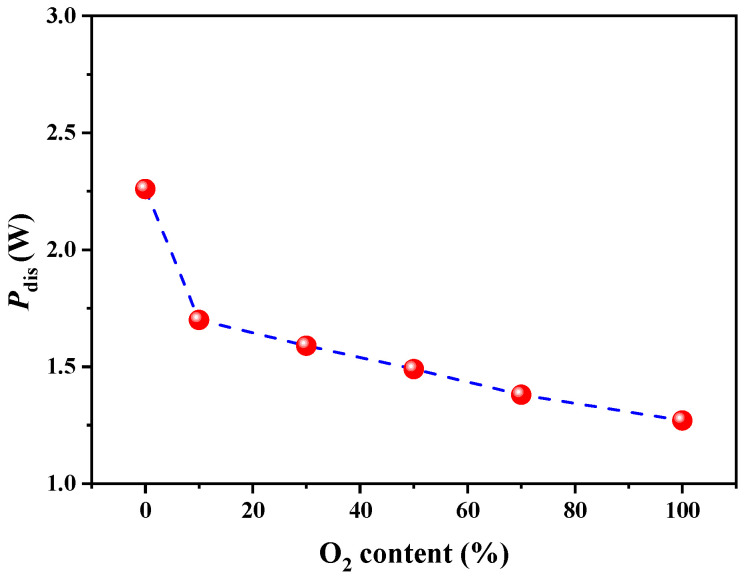
Effect of O_2_ content in O_2_/Ar plasmas on discharge power.

To gain insight into the plasma activation process, the discharge voltage and discharge current in 10% O_2_/Ar, 30% O_2_/Ar and 50% O_2_/Ar are compared in Figure 2. All the waveforms show a typical filamentary DBD mode in which numerous intense current pulses of micro-discharge appear per half voltage cycle [34,35]. When the O_2_ content is 10%, the number of current pulses generated by the discharge is large, and the pulse amplitude is small. This result indicates that discharge in 10% O_2_/Ar plasma is relatively uniform and similar to a typical Ar discharge. As the O_2_ content increases at a fixed input power, the number of current pulses decreases, and the pulse amplitude increases. The waveforms of the 50% O_2_/Ar plasma are close to that of O_2_ discharge. The influence of O_2_ content on the discharge current can be attributed to the increasing excitation channels, which include dissociation, vibration and rotation of O_2_ molecules, requiring more energy. The electronegativity is enhanced to form more negative ions with increasing O_2_ content. The channel contraction and the intensity of micro-discharge channels strengthen [36]. The dependence of discharge power on O_2_ content is also illustrated in Figure 3. A sharp decrease of discharge power occurs from 2.2 to 1.7 W with the O_2_ content increasing from 0% to 10%. The power declines very slowly as the O_2_ content increases from 10% to 50%. The addition of O_2_ in Ar gas leads to an increase in the intensity of the micro-discharge pulse but a decrease in the number of current pulses, resulting in a decrease in discharge power [37,38]. This means that the efficiency of electrical energy transferred to plasma is weakened with increasing O_2_ and that the number of activated Au/TiO_2_ particles decreases in one cycle.

### 3.2. Photocatalytic Performance

**Figure 4:**
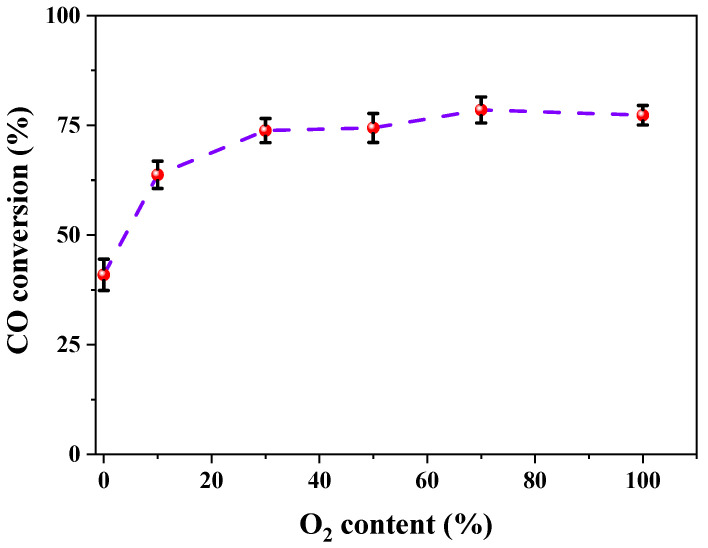
Effect of O_2_ contents in O_2_/Ar discharge gas on CO conversion.

To investigate the effect of the O_2_/Ar ratio on the visible-light photocatalytic performance, the variations in CO conversion over the samples that were treated with pure Ar, 10% O_2_/Ar, 30% O_2_/Ar, 50% O_2_/Ar, 70% O_2_/Ar and pure O_2_ as discharge gases are illustrated in Figure 4. The CO conversion rises rapidly from 40.9% to 63.7% as the O_2_ content increases from 0% to 10%, and reaches 73.8% at 30% O_2_/Ar. There is a weak dependence of CO conversion on O_2_ content as it exceeds 50%. This result proves that O_2_ plays a vital role in the activation of Au/TiO_2_. The reason is that Au/TiO_2_ activated by O_2_ has more surface oxygen, and the high content of surface oxygen generated in O_2_ plasma favors superoxide (O_2_^−^) formation by accepting hot electrons to promote CO oxidation [25,27,32,33]. However, it is worth noting that the O_2_ content in O_2_/Ar plasma is not proportional to the photocatalytic activity. To understand the relationship between physicochemical properties and photocatalytic activity of samples in O_2_/Ar plasma, various characterizations, such as UV-vis DRS, TEM, XPS and CO chemisorption, were carried out.

### 3.3. Optical Property

**Figure 5:**
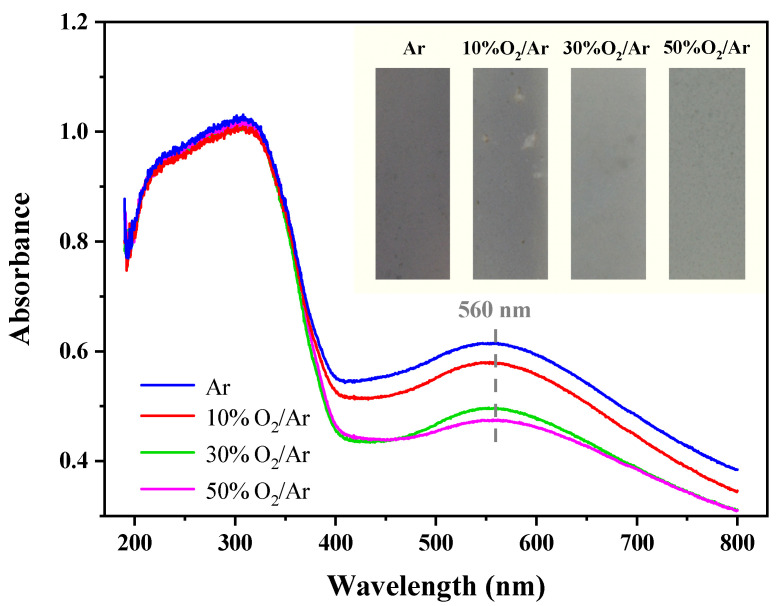
UV-vis DRS of Au/TiO_2_ samples activated by Ar plasma, 10% O_2_/Ar, 30% O_2_/Ar and 50% O_2_/Ar plasmas. Inset photos of Au/TiO_2_ samples activated at various O_2_ contents.

In the visible-light photocatalytic reaction, visible-light absorption of Au/TiO_2_ is based on the surface plasmon resonance effect of Au^0^. To investigate the optical properties, UV-vis DRS of Au/TiO_2_ activated by Ar, 10% O_2_/Ar, 30% O_2_/Ar and 50% O_2_/Ar was measured, as shown in Figure 5. The absorption band of all samples in the visible-light region is centered at 560 nm [39,40], which is attributed to the LSPR absorption bands of Au^0^. The Au/TiO_2_ sample activated by Ar plasma has the strongest LSPR absorption peak, inferring the highest content of Au^0^. As the O_2_ content in the discharge gas increases, the LSPR absorption peaks become weak and present a slight difference in peak intensity between samples activated by 30% O_2_/Ar and 50% O_2_/Ar. This result is consistent with the inset photos shown in Figure 5. The color of the Au/TiO_2_ sample activated by Ar plasma appears light purple [39,41] and becomes light with increasing O_2_ content. This is because Au^0^ nanoparticles absorb green light at approximately 560 nm, and the complementary color is purple. The decreasing Au^0^ content leads to a lighter purple color. It is suggested that the reduction of cationic Au occurs easily in Ar plasma but is depressed with the addition of O_2_.

### 3.4. TEM Observation

**Figure 6:**
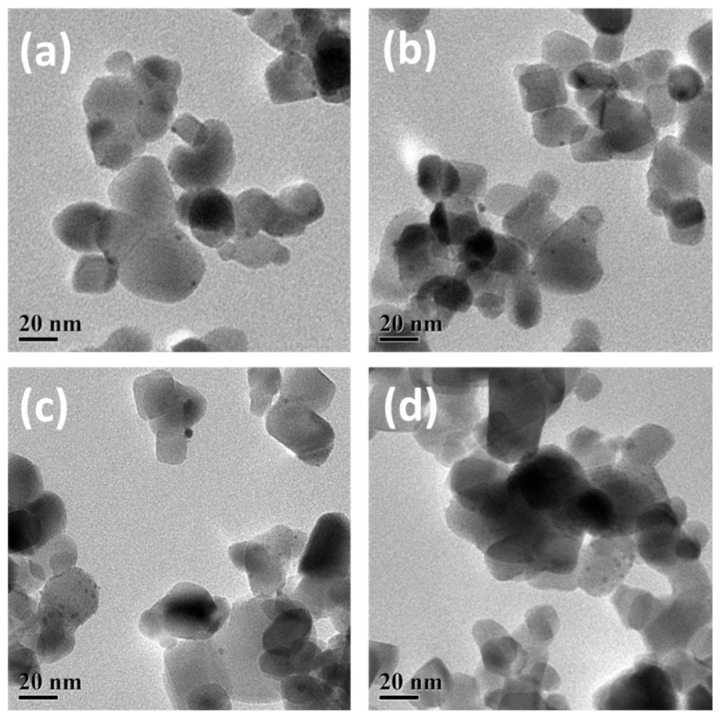
TEM images and the associated particle size histograms of the Au/TiO_2_ samples activated by (**a**) pure Ar, (**b**) 10% O_2_/Ar, (**c**) 30% O_2_/Ar and (**d**) 50% O_2_/Ar plasmas.

It is well-known that the size of Au nanoparticles has a great influence on the photocatalytic activity [42,43,44]. By TEM, the size of Au nanoparticles was observed, as shown in Figure 6. The majority of diameters of Au nanoparticles for samples activated by pure Ar, 10% O_2_/Ar, 30% O_2_/Ar and 50% O_2_/Ar plasmas are around 2 nm. It indicates that the moderate operation of O_2_/Ar cold plasma inhibits the aggregation of Au nanoparticles and that the O_2_ content is independent of the size of the Au nanoparticles. Meanwhile, the size distribution of Au nanoparticles slightly decreases as the O_2_ content rises. This is because the negative charges in the O_2_/Ar plasma sheath could establish a Coulomb field over the surface of Au nanoparticles to remain highly dispersed.

### 3.5. Surface Chemical State Analysis

**Figure 7:**
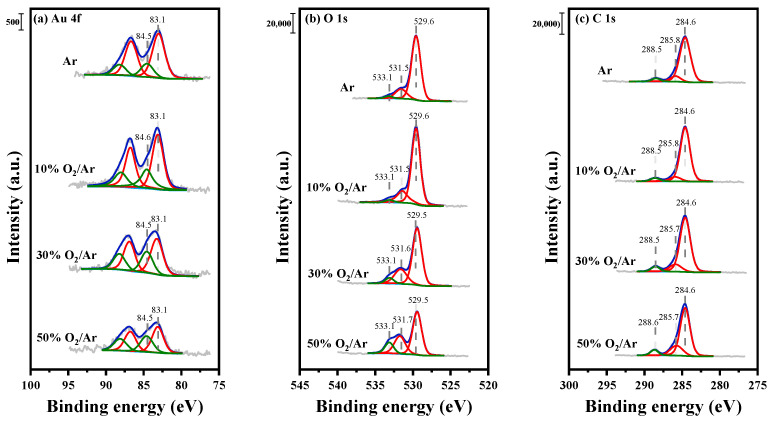
XPS spectra of the samples activated by plasmas at various O_2_ contents in O_2_/Ar discharge gas: (**a**) Au 4f, (**b**) O 1s and (**c**) C 1s.

The high-resolution XPS measurements for three samples were characterized to analyze their surface chemical state. XPS spectra of Au 4f, O 1s and C 1s are shown in Figure 7, and the corresponding results of the samples are summarized in Table 1. The chemical valences of Au, surface oxygen and carbonate species were the focus. After fitting analysis, as shown in Figure 7, the XPS spectra of Au 4f can be deconvoluted into Au^0^ at ~83.1 eV and Au^+^ at ~84.6 eV [27]. As shown in Table 1, the metallic Au content of the samples decreases from 68.0% to 60.2% with increasing O_2_ contents, while the cationic Au content rises, demonstrating that the addition of O_2_ suppresses the reduction of cationic Au. This result is consistent with the intensity sequence of the LSPR peak in UV-vis DRS (Figure 5). The amount of surface oxygen species has a great influence on the photocatalytic activity of CO oxidation. The XPS spectra of O 1s shown in Figure 7b are fitted with three peaks at ~529.5, ~531.6 and ~533.1 eV [45,46], corresponding to the crystal lattice oxygen of TiO_2_, surface oxygen and adsorbed H_2_O or carboxyl groups [47,48], respectively. The content of surface oxygen species increases obviously owing to the change in the oxidative atmosphere listed in Table 1. When the O_2_ content exceeds 30%, the content of surface oxygen slightly increases. The intensity of the peak at ~533.1 eV attributed to C-O species followed a strong dependence on O_2_ content. In the C 1s spectra, the peaks at ~285.8 and ~288.5 eV are assigned to C-O and C=O [49,50], which also increase with the O_2_ content, suggesting that adsorbed carboxyl groups were possible on the surface at a higher O_2_ content.

### 3.6. CO Chemisorption

**Figure 8:**
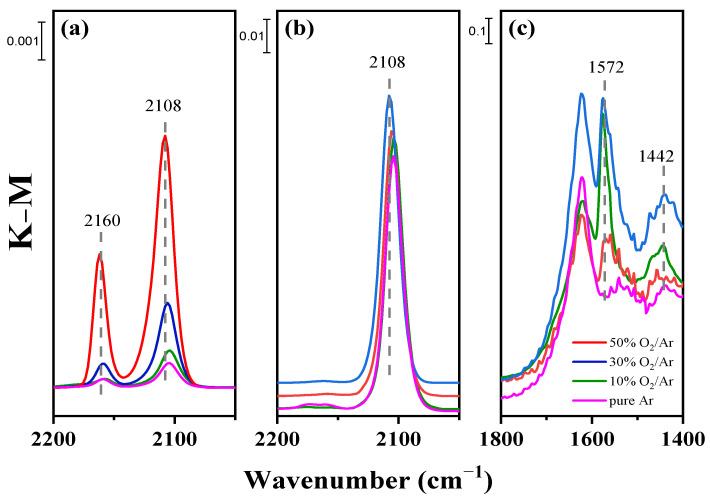
In situ DRIFT spectra of CO adsorption at exposure times of (**a**) 2 min at the band of 2050~2200 cm^−1^, (**b**) 7 min at the band of 2050~2200 cm^−1^ and (**c**) 7 min at the band of 1400~1800 cm^−1^.

With respect to surface species identification, the chemisorption behavior of the CO reactant on the three samples was determined. The evolution of DRIFT spectra of CO adsorbed on Au/TiO_2_ at room temperature is shown in Figure 8. At the initial time (2 min), all samples show two bonds: one is attributed to CO adsorbed on Au^0^ at 2108 cm^−1^ [51,52], and the other is the band near 2160 cm^−1^ [53,54] attributed to CO adsorbed on Au^+^. The band of the sample activated by 50% O_2_/Ar plasma attributed to CO adsorbed on Au^0^ is highest among the samples. This means that CO chemisorption immediately occurs on the Au nanoparticles where surface oxygen species are abundant. This is because samples with a large number of surface oxygen species provide more electron-deficient metallic gold sites for CO chemisorption [26,55]. At a long exposure time (7 min), only the band attributed to CO adsorbed on Au^0^ can be found. The band of CO adsorbed on Au^+^ disappeared along with the significant intensification of CO adsorbed on Au^0^, indicating that the reduction of cationic Au to metallic Au quickly occurs in the CO reaction. In addition, the heights of the bands of CO adsorbed on Au^0^ for the samples are the same, inferring that the number of adsorption sites on the samples is approximate, which agrees with the result of Au nanoparticle size in the TEM images. In Figure 8c, the carbonate species whose bands are near 1420 and 1570 cm^−1^ [56,57] increase with the O_2_ content, indicating that surface oxygen species on catalysts react with adsorbed CO to generate CO_2_ escaped from the surface, or that some carbonate species remain during the CO chemisorption process. It can be speculated that the sites of surface oxygen species adsorbed on Au/TiO_2_ should be different.

In summary, the addition of O_2_ in Ar gas would destroy the homogenous discharge, and negative charges in the O_2_/Ar plasma sheath could establish a Coulomb field on the surface of Au nanoparticles to prevent aggregation. The average diameter of Au nanoparticles is approximately 2 nm, as observed in TEM images (Figure 4), and Au/TiO_2_ activated by plasmas at various O_2_/Ar ratios presents approximate amounts of CO chemisorption. During plasma activation, O_2_/Ar plasma prevents cationic Au from reduction and generates more active surface oxygen species on catalysts, as measured by XPS (Figure 7). The active surface oxygen can be reduced into superoxide by hot electrons to participate in CO oxidation, which follows a Langmuir–Hinshelwood mechanism. This means that a catalyst that possesses a large amount of active surface oxygen is sure to have a high activity at the same CO adsorption capacity. The amount of surface oxygen species increases with the O_2_ content, but CO conversion remains at 75% as the O_2_ content exceeds 50%. Combining the C 1s spectra in XPS and CO chemisorption over the samples, it can be deduced that there are two sites of active surface oxygen. One is around the interface between Au NPs and TiO_2_ support, and the other is on the surface of TiO_2_. The interaction between micro-discharges and Au/TiO_2_ increases as the O_2_ content increases. That is, surface oxygen on TiO_2_ is generated and remains stable. The oxygen species around the interface between Au NPs and TiO_2_ support engage in CO oxidation to promote the performance. The oxygen species on the TiO_2_ support form carbonate species, which are irrelevant to photocatalytic activity. As a result, as the O_2_ content increases, CO conversion rises rapidly and then slightly.

## 4. Conclusions

The effect of O_2_ content in O_2_/Ar plasma on the activation of Au/TiO_2_ was studied. The number of current pulses decreased, and the pulse amplitude increased with the O_2_ content. However, discharge power dropped quickly. CO conversion over Au/TiO_2_ rose rapidly at first and then remained constant once the O_2_ content reached 50%. Compared with the discharge gas of 10% and 30% O_2_/Ar, the sample activated by 50% O_2_/Ar plasma possessed less metallic Au and more surface oxygen species and carbonate species. The CO chemisorption capacities of samples activated by 10%, 30% and 50% O_2_/Ar plasmas were almost the same at a long exposure time due to the approximate Au nanoparticle size shown in TEM. The content of surface oxygen species slightly increased when the O_2_ content was over 50%. Carbonate species were generated from the oxygen species on the surface of TiO_2_ according to the analysis of XPS and CO chemisorption.

## Figures and Tables

**Table 1:** XPS analysis of the Au/TiO_2_ samples.

Samples	Proportion (at.%)				
	O_surf_ (~531.6 eV)/O	Au^0^/Au	Au^+^/Au	C-O/C	C-OO/C
Ar	14.4	68.0	32.0	32.0	
10% O_2_/Ar	16.1	67.4	32.6	8.6	5.3
30% O_2_/Ar	19.6	63.3	36.7	11.9	5.9
50% O_2_/Ar	20.0	60.2	39.8	16.3	7.0
